# Supplementary material for: Deep flanking sequence engineering for efficient promoter design using DeepSEED
Source: Nat Commun. 2023 Oct 9;14:6309. doi: 10.1038/s41467-023-41899-y (PMC10562447; doi:10.1038/s41467-023-41899-y)
Supplement: Supplementary file 1 — Supplementary Information [file 41467_2023_41899_MOESM1_ESM.pdf]

Supplementary information

Deep flanking sequences engineering for efficient promoter design using DeepSEED

Supplementary Notes

DeepSEED network structure

Two networks, conditional generative adversarial model and promoter activity prediction model, were constructed for promoter design (Supplementary Fig. 1). The precise details of the two networks can be found in the code, or described below.

I. Conditional generative adversarial model (two networks, generator and discriminator)

   (1) Generator:
   Input layer (dims = SeqLength * 4)
   Dense (units = 512)
   Attention (embed_dim = 512, heads = 16)
   Resblock (Conv_kernelsize = 13, padding = 6, filters = 512)
   Resblock (Conv_kernelsize = 13, padding = 6, filters = 512)
   Resblock (Conv_kernelsize = 13, padding = 6, filters = 512)
   Resblock (Conv_kernelsize = 13, padding = 6, filters = 512)
   Resblock (Conv_kernelsize = 13, padding = 6, filters = 512)
   Conv (Conv_kernelsize = 1, padding = 0, filters = 4)
   Softmax (dims = 1)

   (2) Discrimininator:
   Input layer (dims = (SeqLength,8))
   Conv (Conv_kernelsize = 1, padding = 0, filters=512)
   Attention (embed_dims = 512, heads = 16)
   Resblock (Conv_kernelsize = 13, padding = 6, filters = 512)
   Resblock (Conv_kernelsize = 13, padding = 6, filters = 512)
   Resblock (Conv_kernelsize = 13, padding = 6, filters = 512)
   Resblock (Conv_kernelsize = 13, padding = 6, filters = 512)
   Resblock (Conv_kernelsize = 13, padding = 6, filters = 512)
   Dense (units = 1)

II. Promoter activity prediction model
   Conv (Conv_kernelsize = 7, padding = 3, filters=64)
   MaxPool (pool_size = 3, strides = 2)
   LSTM (output_size=64, bidirectional = True)
   DenseBlock (block_config=(2, 2, 4, 2), filters=128)
   AveragePool (pool_size = 7, padding = 3, strides = 1)
   Dense (units = 1)

SeqLength represents the length of promoter sequences. Conv represents the convolutional layer. Resblock represents a building block of ResNet proposed by He et al[1]. DenseBlock represents the dense block proposed by Huang et al[2]. Attention represents the multi-head attention layer proposed by Vaswani et al[3].

Train/Test/Validation Set Splitting and DeepSEED performance comparison

To develop the prediction model, we partitioned the samples into three sets: 80% for the training set, 10% for the validation set, and 10% for the test set. This partitioning was applied to both the *E. coli* and mammalian cell predictors. The *E. coli* promoter predictor achieved a high Pearson correlation coefficient (PCC) of 0.74 compared with our previous CNN-based predictive model[4] (PCC = 0.65) in the *E. coli* promoter dataset (Supplementary Fig. 19).

Regarding the training of the generator, as it involves unsupervised learning, we did not apply specific dataset partitioning applied. Instead, we monitored the k-mer frequencies during the training process. Notably, for *E. coli* promoters, we observed a strong correlation between the k-mer frequency (k = 4 to 6) of the natural sequences and the DeepSEED-designed sequences at the global scale, which showed a high PCC compared to the sequences generated by our previous Wasserstein generative adversarial network–gradient penalty (WGAN-GP) model[4] (Supplementary Fig. 4d).

DeepSEED framework for promoter design

The DeepSEED framework was created by two deep learning models (conditional generative adversarial model, activity prediction model) and one optimization algorithm (genetic algorithm). The code is available on GitHub (https://github.com/WangLabTHU/DeepSEED). Briefly, the role of the conditional generative adversarial model is to estimate the conditional probability distribution of functional promoters based on the 'seed' inputs, then the synthetic promoters could be sampled from the conditional distribution. The role of the promoter activity prediction model is to estimate the gene expression activity of promoters. The role of the genetic algorithm is to combine two models to optimize the promoter's activity.

Promoter design happened in three steps: (1) Conditional generative adversarial model was trained by the 'seed' sequences and their corresponding natural promoter sequences in the training dataset (Fig. 1b). (2) Activity prediction model was trained by the natural promoter sequences and their activity in the training dataset (Fig. 1b). (3) After two models were trained, the generator of the conditional generative adversarial model and the activity prediction model were concatenated, in which the output of the generator was used as the input of the activity prediction model (Fig. 1a). Then the genetic algorithm was used to design the synthetic promoters based on the 'seed' sequences. The synthetic sequences obey the functional promoter distribution (learned by conditional generative adversarial model) and will show high activity (learned by activity prediction model) (Fig. 1c).

Details of conditional GANs (cGANs), predictor training and genetic algorithm optimization

cGANs training: Vanilla GANs have been trained on the unsupervised training data, i.e. promoter sequences. During the training, the input of the vanilla generator is the latent vector, and the output of the vanilla generator is the synthetic promoters. The role of the vanilla discriminator is to classify the synthetic and natural promoters. To

integrate expert prior knowledge into promoter design, we used cGANs. During the training, the input of the generator is the ‘seed’ sequences, which is the concatenated vector of the latent vector and seed vector, and the output of the generator is the synthetic promoter (Supplementary Fig. 1, left). Here, the ‘seed’ vector determined the location and nucleobase of the knowledge-defined region, and the latent vector determined the location of the model-defined region. The inputs of the discriminator of cGANs are the sample pair of ‘seed’ sequences and generated/natural sequences, in which the ‘seed’ sequences deliver the expert knowledge to the discriminator (Supplementary Fig. 1, middle). The discriminator is trained five times, whereas the generator is trained one time in each iteration.

Predictor training: During the predictor training, the natural sequences from the training set are set as input, and the mean squared error (MSE) between predictor outputs and their ground truth activities are set as the loss to optimize the predictor.

Genetic algorithm optimization: For the promoter design problem, we used the genetic algorithm to optimize the flanking sequences based on the ‘seed’ assignment. The initial population of the genetic algorithm consists of random vectors. The hybrid networks of the generator (from cGANs) and predictor were used to estimate the fitness score of each seed sequence. The random vectors in the ‘seed’ sequences were used as the initial population, and they were optimized by the genetic algorithm to find the final solutions.

Details of constitutive promoter design in *E. coli*
29248 promoter sequences with 165bp length were collected from Johns et al., and their activities were measured by RNA-seq[5]**.** Promoter sequences were annotated by –10/–35 motifs, then generating seed sequences with the following patterns:
‘NNNNNNNNNNNNNNNNNNNNNNNNNNNNNNNNNNNNNNNNNNNNNNNNNNNN
NNNNNNNNNNNNNNNNNNNNNNNNNNNNNNNNNNNNNNNNNNNNNNNNNNNNN
TTGACANNNNNNNNNNNNNNTATAATNNNNNNNNNNNNNNNNNNNNNNNNNNN
NNNNNNNNN’
11601 sequences with distinct –10 and – 35 regions were chosen from the dataset. Their promoter sequences, ‘seed’ sequences, and activities were used for training cGANs and predictors. For *E. coli* promoter design, we select three template promoters from the iGEM parts registry (BBa_J23119, BBa_J23118, and BBa_J23114, http://parts.igem.org/Promoters/Catalog/Constitutive), and annotated the –10 and – 35 regions, and generating ‘seed’ sequences as follows:
BBa_J23119:
‘**TTGACA**GCTAGCTCAGTCCTAGG**TATAAT**GCTAGC’ (BBa_J23119 original sequences, 35bp)
‘NNNNNNNNNNNNNNNNNNNNNNNNNNNNNNNNNNNNNNNNNNNNNNNNNNNN
NNNNNNNNNNNNNNNNNNNNNNNNNNNNNNNNNNNNNNNNNNNNNNNNNNNNN
NNN**TTGACA**NNNNNNNNNNNNNNNNNN**TATAAT**NNNNNNNNNNNNNNNNNNN
NNNNNNN

NNNNN’ (BBa_J23119 ‘seed’ sequences, 165bp. Random sequences were used to extend the initial promoter sequences to 165bp)

BBa_J23118:

‘**TTGACG**GCTAGCTCAGTCCTAGG**TATTGT**GCT’ (BBa_J23118 original sequences, 35bp)

‘NNNNNNNNNNNNNNNNNNNNNNNNNNNNNNNNNNNNNNNNNNNNNNNNNNN
NNNNNNNNNNNNNNNNNNNNNNNNNNNNNNNNNNNNNNNNNNNNNNNNNNNN
NNN**TTGACG**NNNNNNNNNNNNNNNNNN**TATTGT**NNNNNNNNNNNNNNNNNNN
NNNNNNN
NNNNN’ (BBa_J23118 ‘seed’ sequences, 165bp. Random sequences were used to extend the initial promoter sequences to 165bp)

BBa_J23114:

‘**TTTATG**GCTAGCTCAGTCCTAGG**TACAAT**GCTAGC’ (BBa_J23114 original sequences, 35bp)

‘NNNNNNNNNNNNNNNNNNNNNNNNNNNNNNNNNNNNNNNNNNNNNNNNNNN
NNNNNNNNNNNNNNNNNNNNNNNNNNNNNNNNNNNNNNNNNNNNNNNNNNNN
NNN**TTTATG**NNNNNNNNNNNNNNNNNN**TACAAT**NNNNNNNNNNNNNNNNNNN
NNNNNNN
NNNNN’ (BBa_J23114 ‘seed’ sequences, 165bp. Random sequences were used to extend the initial promoter sequences to 165bp)

The flanking sequences of –10 and – 35 regions were optimized by the genetic algorithm. With 60 iterations of optimization, the synthetic promoters with the best predicted property scores were chosen for experimental validation. The detailed sequences are provided in Supplementary Data 1.

Details of IPTG-inducible promoter design in *E. coli*

Different from constitutive promoter design in *E. coli,* the promoter sequences from Johns et al. were firstly annotated by –10 and – 35 regions, then annotated several regions based on their relative position between lacO sites and –10/–35 regions (Supplementary Fig. 12). For example, if we have 165-bp natural promoter sequence with annotated –10 and – 35 regions, which was shown as follows:

‘GTCAATTTCAACTTAAAACTAATTTTGAAGAAGTCATTCAAAGACATGCTTTATTT
AGAAAGGAACAACAATTCTGGGAAATAATATTTTT**TAGTAA**TTTCAGACAGAATA
TATAGATAAGG**TATAAT**ATTAAATACAAAATTTATATAACAAGGGGGATTATTC’

Then based on the 2lacO, 3lacO, and 4lacO relative position, we have ‘seed’ sequences:

2lacO:

‘NNNNNNNNNNNNNNNNNNNNNNNNNNNNNNNNNN**CATTCAAAGACATGCTTT
ATT**NNNNNNNNNNNNNNNNNNNNNNNNNNNNNNNNNNNN**TAGTAA**NNNNNNN
NNNNNNNNNNNNNNNNNN**TATAAT**NNNNNN**TACAAAATTTATATAACAAGG**NN
NNNNNNNN’

3lacO:

‘NNNNNNNNNNNNNNNNN**ACTAATTTTGAAGAAGTCATTCAAAGACATGCTTTA
TT**NNNNNNNNNNNNNNNNNNNNNNNNNNNNNNNNNNNN**TAGTAA**NNNNNNNN

NNNNNNNNNNNNNNNNNN**TATAAT**NNNNNN**TACAAAATTTATATAACAAGG**NNN NNNNNNN'

4lacO:

'NNN**AATTTCAACTTAAAACTAATT**NNNNNNNNNN**CATTCAAAGACATGCTTTATT**NNNNNNNNNNNNNNNNNNNNNNNNNNNNNNNNNNNNNNNNN**TTCAGANAGAATATATAGATAAGGTATAATNNNNNNTACAAAATTTATATAACAAGG**NNN NNNNNNN'

To satisfy the relative position constraint, the training samples of 2lacO, 3lacO, and 4lacO are 8082, 5920 and 3354 respectively. After training the models, 'seed' sequences that contain lacO sites at the same location as the training samples are generated, which were shown as follows:

2lacO:

NNNNNNNNNNNNNNNNNNNNNNNNNNNNNNNNNNNNNN**AATTGTTATCGGATAACAATT**NNNNNNNNNNNNNNNNNNNNNNNNNNNNNNNNNNNNNNNNN**CTTACT**NNN NNNNNNNNNNNNNNNNNNNN**TAAAAT**NNNNNN**AAATTGTGAGCGCTCACAATT**N NNNNNNNN (AATTGTTATCGGATAACAATT, AAATTGTGAGCGCTCACAATT: lacO sites)

3lacO:

NNNNNNNNNNNNNNN**AATTGTGAGCGGATAACAATTGGCAGTGAGCGCAACGCAATT**NNNNNNNNNNNNNNNNNNNNNNNNNNNNNNNNNNNNNNNNNNNNNNNNN**TTTAAA**NNNNNNNNNNNNNNNNNN**TATACT**NNNNNN**AAATTGTGAGCGCTCACAATT**N NNNNNNNN

(AATTGTGAGCGGATAACAATTGGCAGTGAGCGCAACGCAATT: two lacO sites)

4lacO:

NNNNNNNNNN**AATTGTGAGCGGATAACAATT**NNNNNNNNNN**AATTGTTATCGGATAACAATT**NNNNNNNNNNNNNNNNNNNNNNNNNNNNNNNNNNNNNNNNNNNNN**ATGACT**NNNNN**TTGTGAGCGGATAACAATAAAAT**NNNNNN**AAATTGTGAGCGCTCACAATT**NNN

The flanking sequences of –10, – 35 regions and lacO sites were optimized by genetic algorithm. With 100 iterations of optimization, the synthetic promoters with the best predicted property scores were chosen for experimental validation. The detailed sequences are provided in Supplementary Data 1.

Details of Dox-inducible promoter design in mammalian cells

Two different datasets were prepared for cGANs[6] and predictor training[7], respectively. HEK293 enhancer dataset accumulated by Wang et al.[6] was used to train the cGANs. 26604 sequences with 150-bp length were trimmed from the active enhancer region provided in the dataset. These sequences were annotated by 1,205 motifs from JASPAR 2022 vertebrate datasets[8], then generating 'seed' sequences with the following patterns:

'**GTTCCCTG**NNNNNNNNNNNNNNNNNNNNNNNNNNNNNN**AGGCAGGGAGAGGCTGGGCTG**NNNNNNNNNNNNNNNNNNNN**CCTGCTGGGTGTTGCCTAGGAGAGGAGAAAAAGCCCCGACGCCAGAAATGGA**NNNNNNNNNNNNN**ACCTCGGAGGG**'

where ‘GTTCCCTG’, ‘AGGCAGGGAGAGGCTGGGCTG’, ‘CCTGCTGGGTGTTGCCTAGGAGAGGA GAAAAAGCCCCGACGCCAGAAATGGA’ and ‘ACCTCGGAGGG’ patterns represent motif sequences, and ‘N’ represents flanking regions of motifs. These annotated ‘seed’ sequences and their enhancer sequences were used for training the cGANs. To train the predictor, 328, 795 sequences and their transcriptional activities provided by Ernst et al.[7] were prepared.

After training the cGANs and predictor, 3tetO ‘seed’ sequences obtained by expert knowledge were prepared as follows:

‘NNNNNNNNNNNNNNNNNNNNNNNNNNNNNNNNNNNNNNNNNNNNNNNNN**TCCCTATCAGTGATAGAGA**NNNNNNNNNNNNNNNN**TCCCTATCAGTGATAGAGAN**NNNNNNNNNNNNNNNN**TCCCTATCAGTGATAGAGA**NNNNNNNNNNN’

(TCCCTATCAGTGATAGAGA: tetO motif)

With 100 iterations of optimization, the synthetic promoters with the best predicted property scores were chosen for experimental validation. The detailed sequences are provided in Supplementary Data 1.

Supplementary Figures

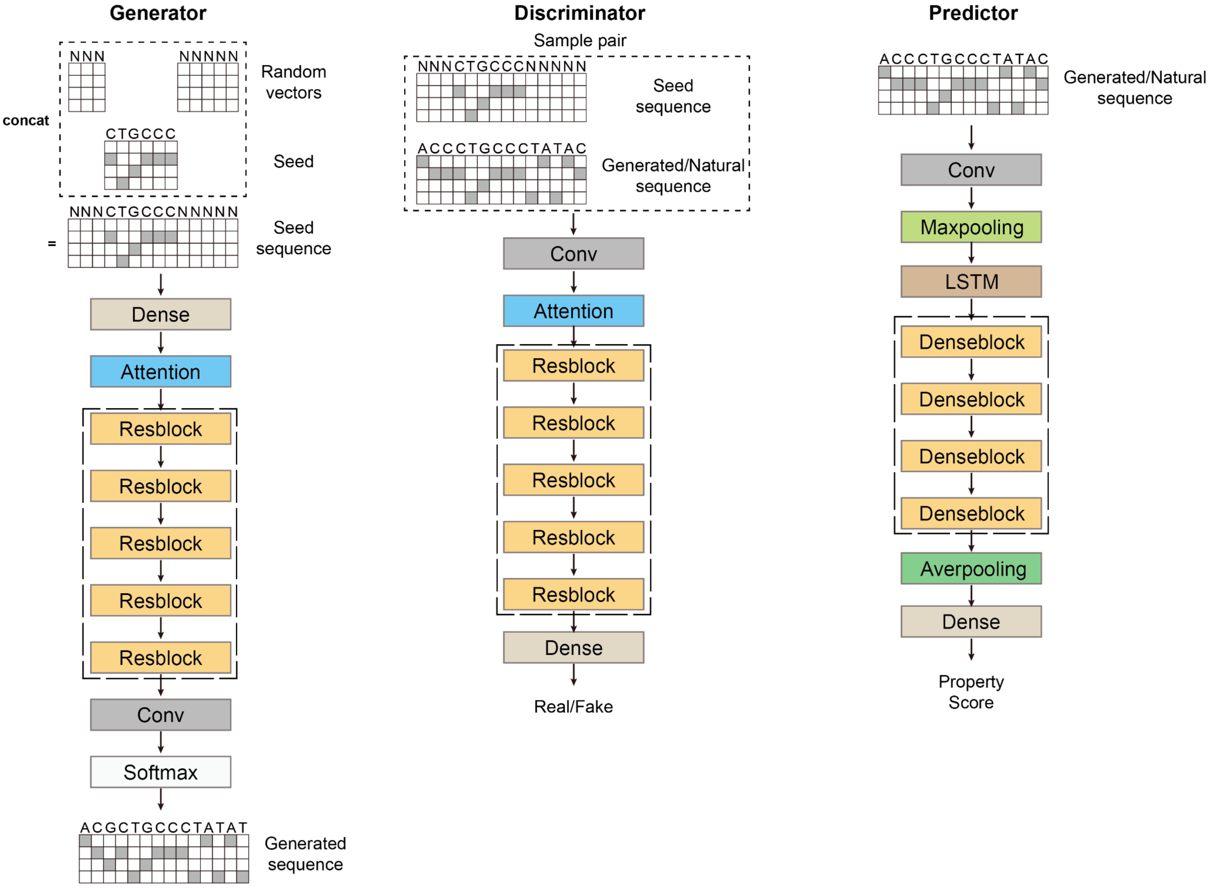

Supplementary Figure 1. The structure of the cGANs model and predictor model. From left to right, the generator of cGANs, the discriminator of cGANs, and the predictor were presented.

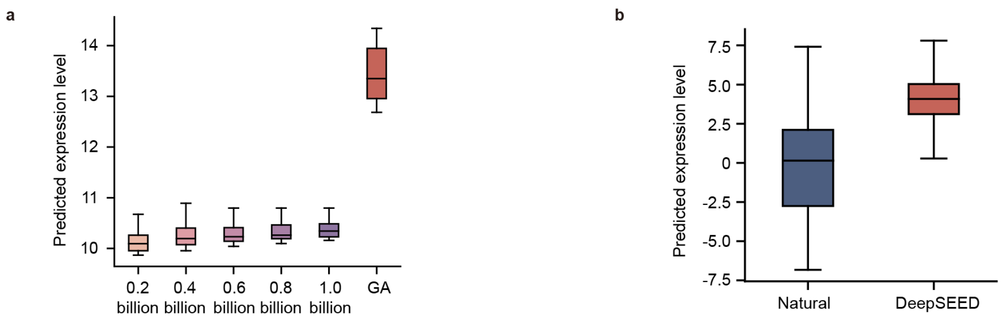

Supplementary Figure 2. a The performance comparison between random sampling and genetic algorithm. The generator model directly generated 0.2 billion to 1 billion sequences and We selected 100 sequences with the highest expression of the predictor output. As a comparison, 100 sequences were generated with the genetic algorithm by the generator. **b** Constitutive promoter design task proved *in silico*. We randomly chose the dataset promoters and optimized their flanking sequence by preserving –10 and –35 regions as expert knowledge. The optimization process same as the constitutive promoter design in Supplementary Notes. The predictor was used to evaluate the promoter activity. Box plots indicate the median (middle line), 25th, 75th percentile (box) and minimum and maximum values (whiskers).

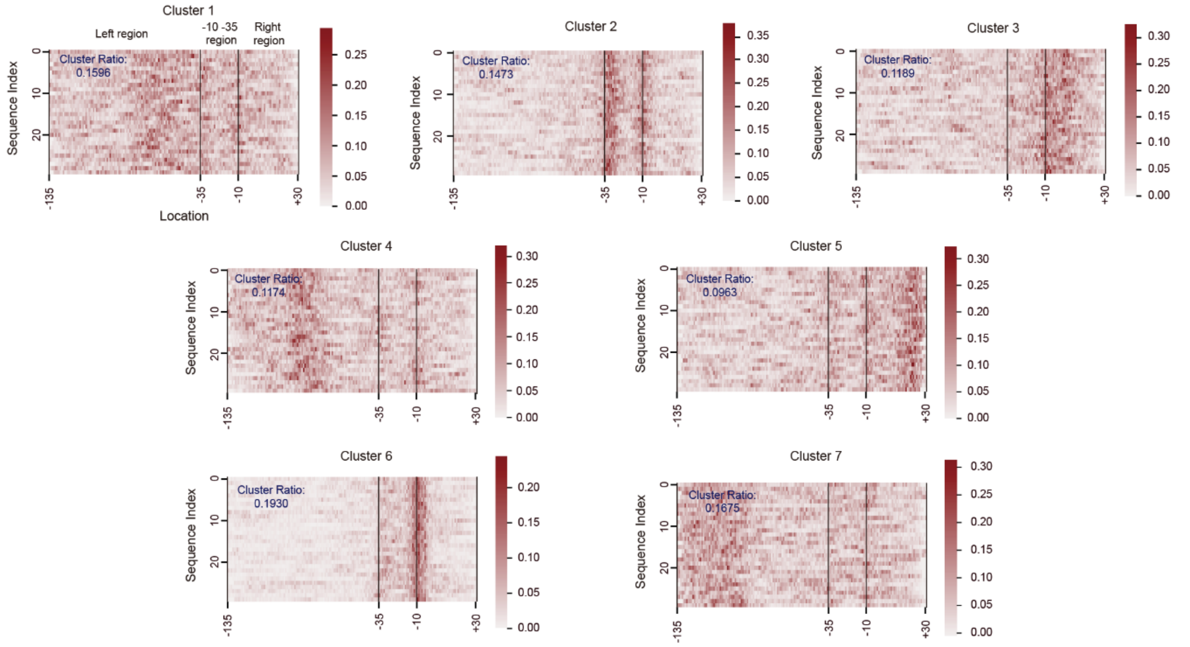

Supplementary Figure 3. Heatmap of gene expression influential values of each location in seven promoter clusters, with each column representing different sequence samples and each row representing nucleotide location corresponding to TSS. Source data are provided as a Source Data file.

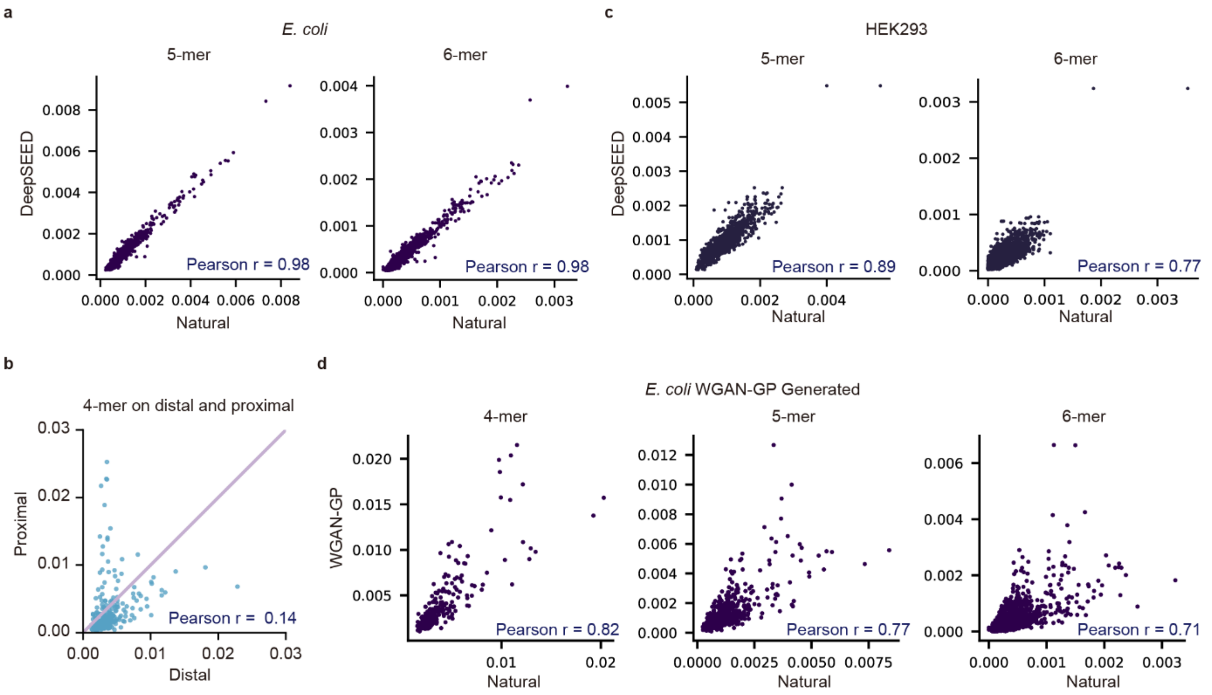

Supplementary Figure 4. a Scatter plot showing 5-mer and 6-mer frequency between natural and DeepSEED generated *E. coli* sequences. **b** Scatter plot showing 4-mer frequency between the proximal and distal region in natural *E. coli* sequences. **c** Scatter plot showing 5-mer and 6-mer frequency between natural and DeepSEED generated Dox-inducible sequences. **d** Scatter plot showing 4-mer to 6-mer frequency between natural and WGAN-GP model[4] generated *E. coli* sequences. Source data are provided as a Source Data file.

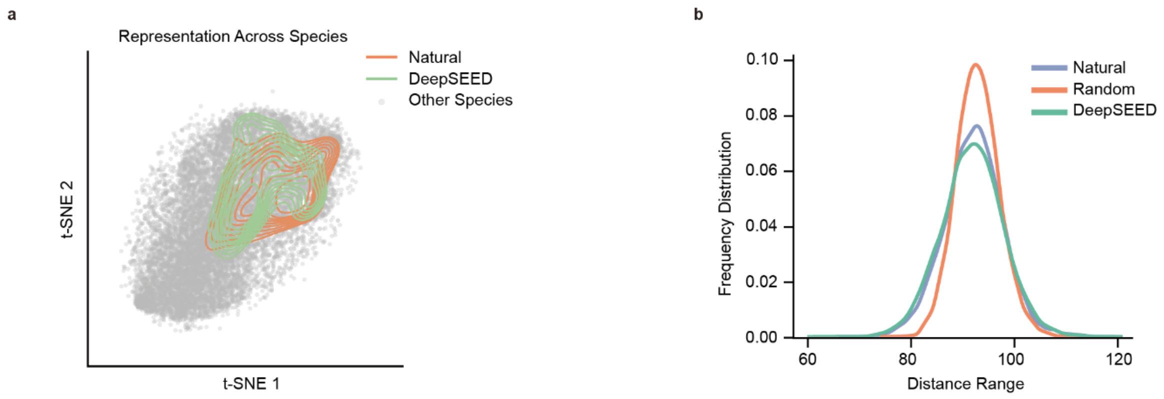

Supplementary Figure 5. a Sequence space of functional sequences in *E. coli* and other species. The green contour lines and orange contour lines showed the distributions of natural sequences and DeepSEED-generated sequences respectively. **b** The frequency distribution of edit distance of natural sequences, random sequences, and DeepSEED-generated sequences. Source data are provided as a Source Data file.

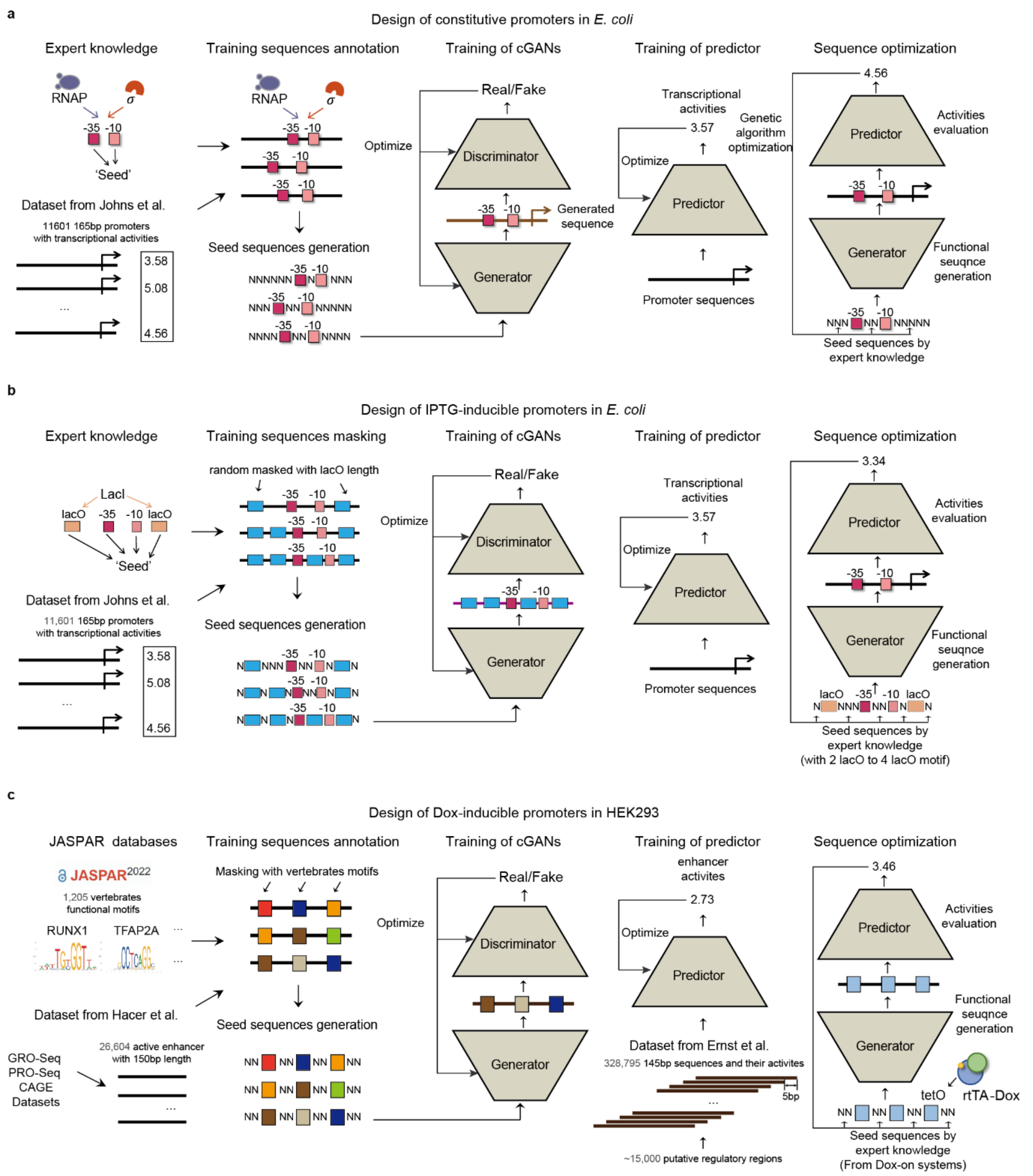

Supplementary Figure 6. The details of the sequence construction in three promoter design tasks during the model training process. **a** Design of constitutive promoters in *E. coli*. **b** Design of IPTG-inducible promoters in *E. coli*. **c** Design of Dox-inducible promoters in mammalian cells.

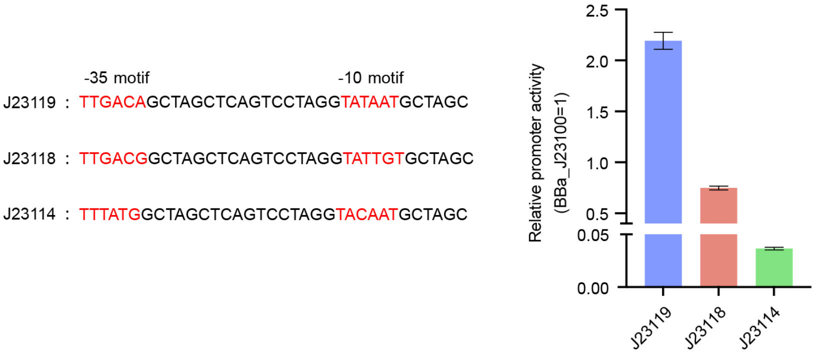

Supplementary Figure 7. The sequence and expression level of BBa_J23 promoters in *E. coli* cell. Three BBa_J23 promoters have different -10 and -35 pairs showing a significant difference in promoter activity. The error bar represents the standard deviation of three biological replicates. Source data are provided as a Source Data file.

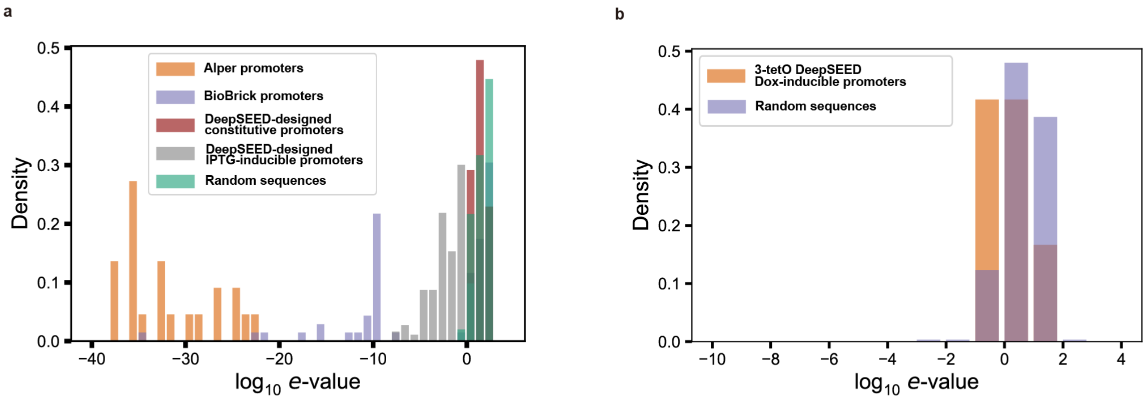

Supplementary Figure 8. The distribution of BLAST e-values for the comparison of promoter sequences with the Escherichia coli K-12 (taxid:83333, **a**) and Home sapiens (taxid:9606, **b**). **a** The BLAST e-value distribution of DeepSEED-designed constitutive promoters, DeepSEED-designed IPTG-inducible promoters, randomly generated sequences, designed promoters from Alper et al[9] and constitutive promoters in iGEM BioBrick (http://parts.igem.org/Promoters/Catalog/Constitutive) standard parts[10]. **b** The BLAST e-value distribution of 3-tetO DeepSEED Dox-inducible promoters and randomly generated sequences. The e-value of the DeepSEED-designed promoters and random sequences are at the same level and show lower similarity with the natural genome than the promoters designed by Alper et al and most constitutive promoters from iGEM BioBrick standard parts. The detailed sequences and results are provided in Supplementary Data 3. Source data are provided as a Source Data file.

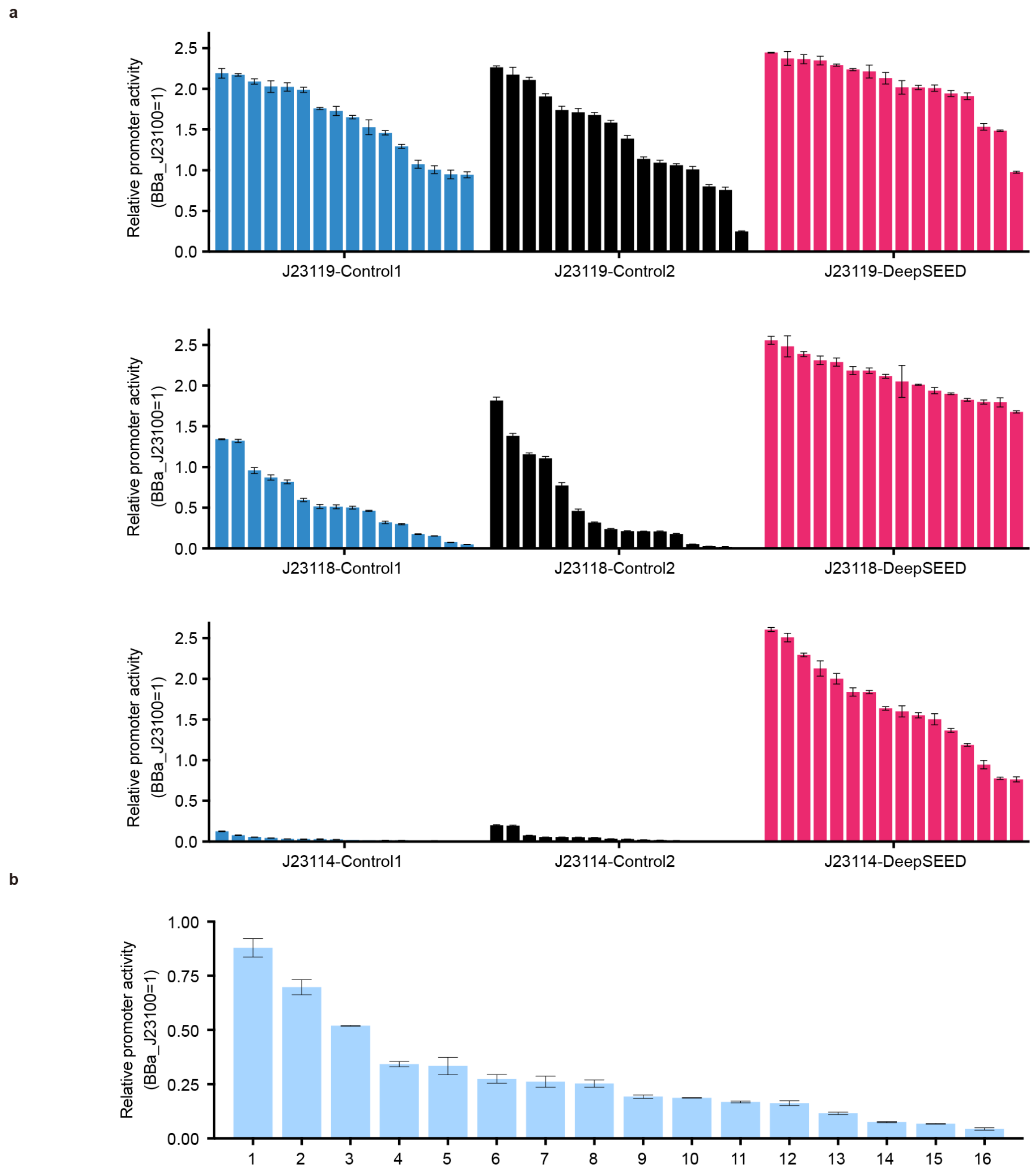

Supplementary Figure 9. a The expression level of the promoters in the constitutive promoter task *in vivo*. The detailed results of Fig. 3c. **b** The expression level of the promoters generated by the NAR2020 model *in vivo*. The detailed results of Fig. 3d. The error bar represents the standard deviation of three biological replicates. Source data are provided as a Source Data file.

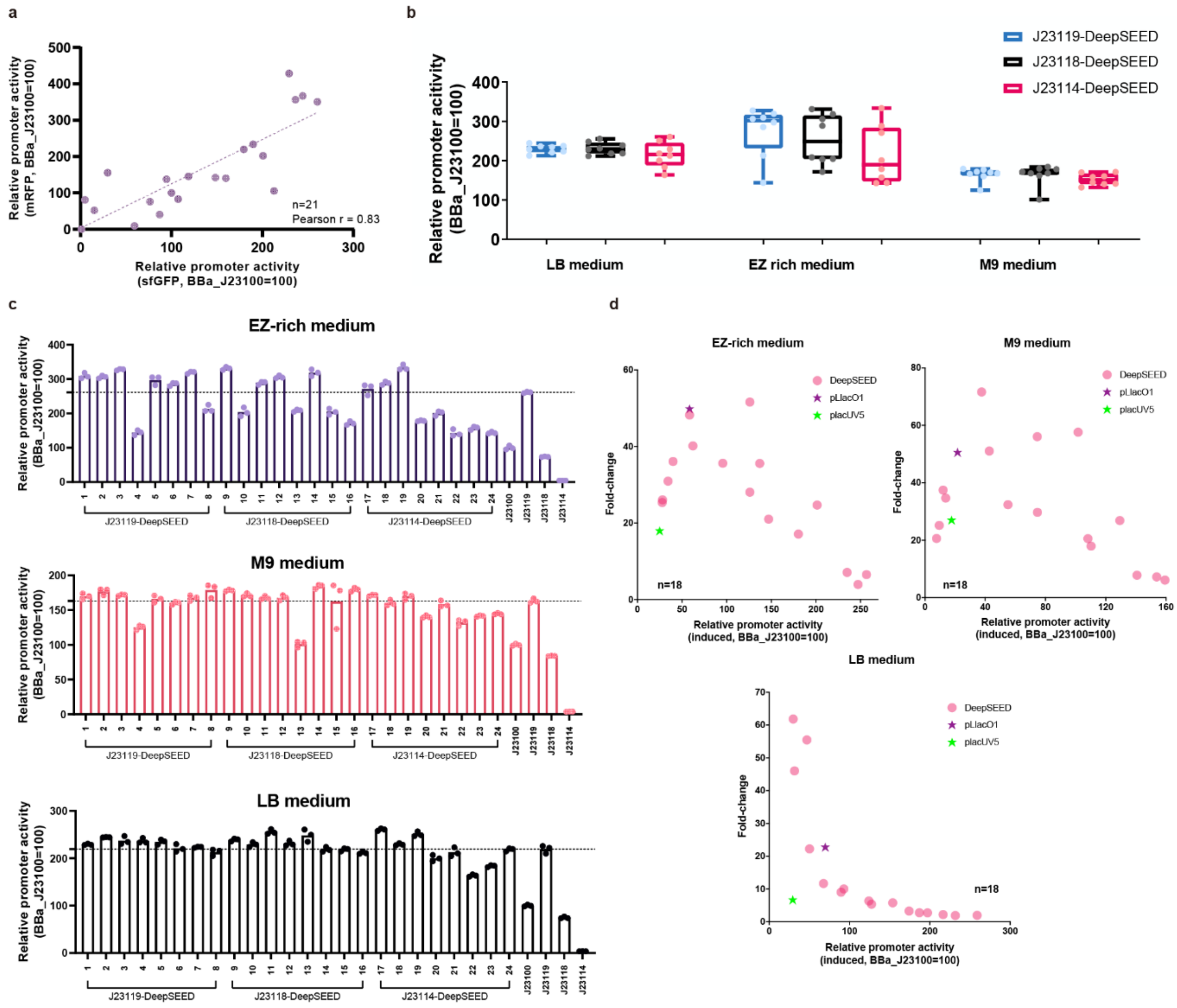

Supplementary Figure 10. a The relative promoter activity of 21 constitutive promoters was evaluated using the reporter gene *mrfp* and *sfgfp* in the constitutive promoter design task**. b-c** A subset of the DeepSEED-designed constitutive promoters was tested in two additional types of culture medium: M9 and EZ-rich. Boxplot showing the promoter activity of the DeepSEED-generated promoter in three initial promoter tasks (**b**). Histogram showing the activity of each promoter in comparison to the short initial promoters (**c**). **d** A subset of the DeepSEED-designed IPTG-inducible promoters was tested in two additional types of culture medium: M9 and EZ-rich. Normalization of promoter activity with strong constitutive promoter J23100 from the iGEM parts registry. Each dot represents the average of three biological replicates. Detailed sequences and results can be found in Supplementary Data 1. Box plots indicate the median (middle line), 25th, 75th percentile (box) and minimum and maximum values (whiskers). Source data are provided as a Source Data file.

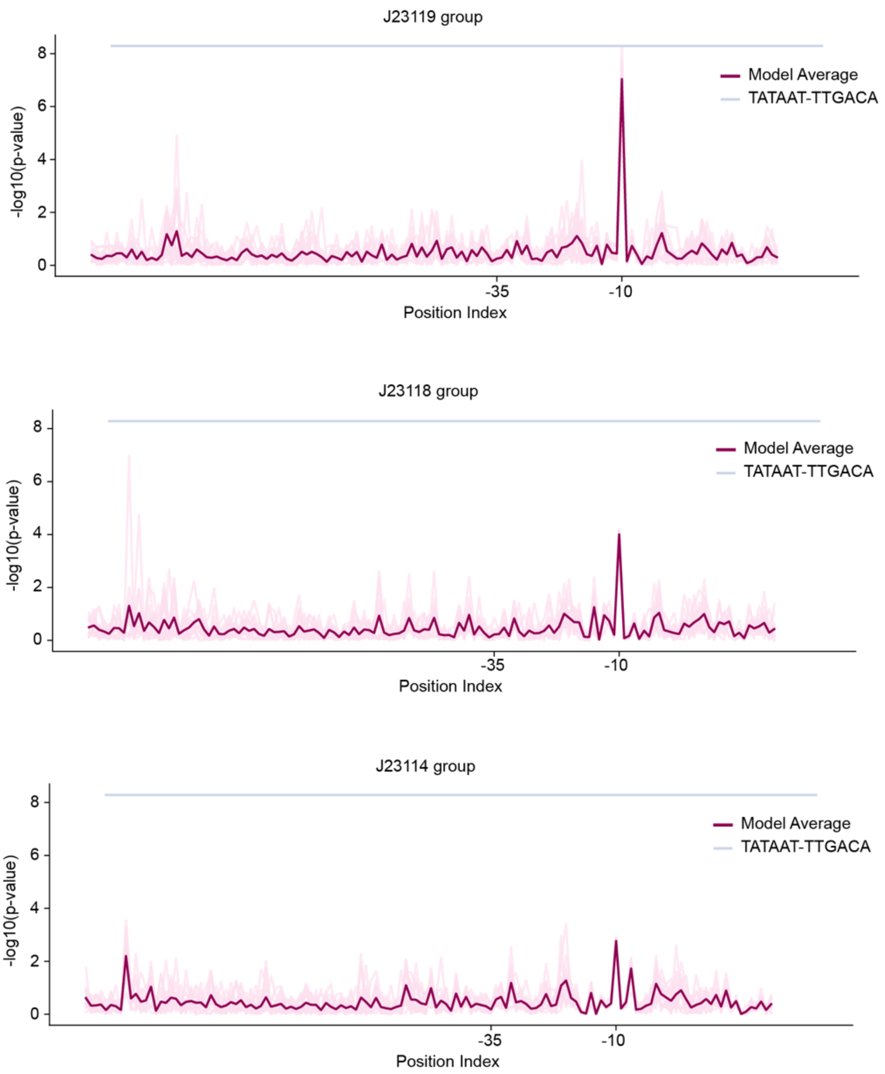

Supplementary Figure 11. Second promoter analysis of DeepSEED-generated promoters in the constitutive promoter task. The blue line, pale red line, and dark red represent the score of the ‘TATAAT-spacer-TTGACA’ structure, each DeepSEED-generated promoter, and the average of the DeepSEED-generated promoters. Source data are provided as a Source Data file.

a

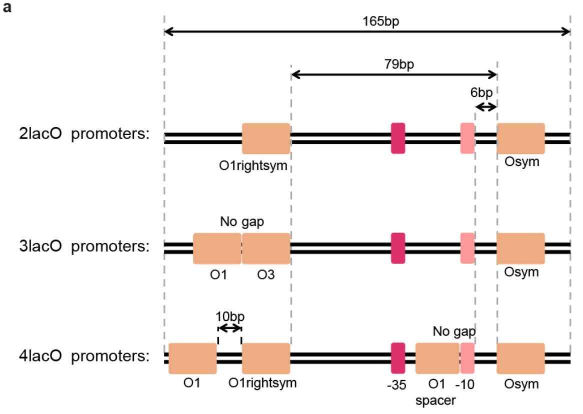

b

Operator Variant	Sequence (5’ to 3’)
Osym	AAATTGTGAGCGCTCACAATT
O1rightsym	AATTGTTATCGGATAACAATT
O1	AATTGTGAGCGGATAACAATT
O3	GGCAGTGAGCGCAACGCAATT
O1 spacer	TTGTGAGCGGATAACAA

Supplementary Figure 12. The details of the IPTG-inducible promoters. **a** The lacO sites position of substitution and model groups (Fig. 4b) in three IPTG-inducible promoter design tasks. **b** The lacO sequences used in this work, which according to a previous study[11].

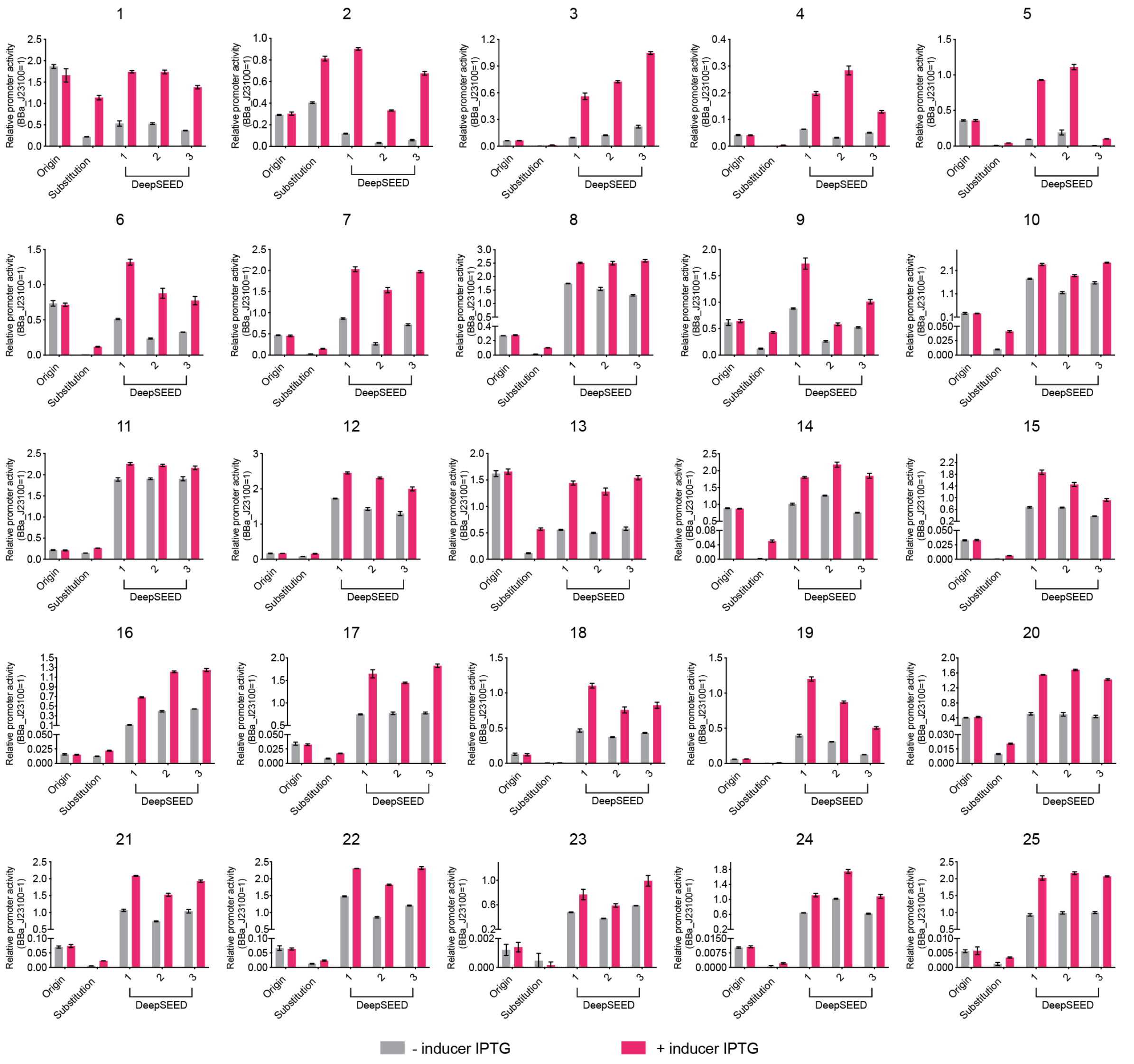

Supplementary Figure 13. The expression level of the 2lacO promoters in the IPTG-inducible promoter task in *E. coli*. The details of each promoter activity under the induced and non-induced conditions. The error bar represents the standard deviation of three biological replicates. Source data are provided as a Source Data file.

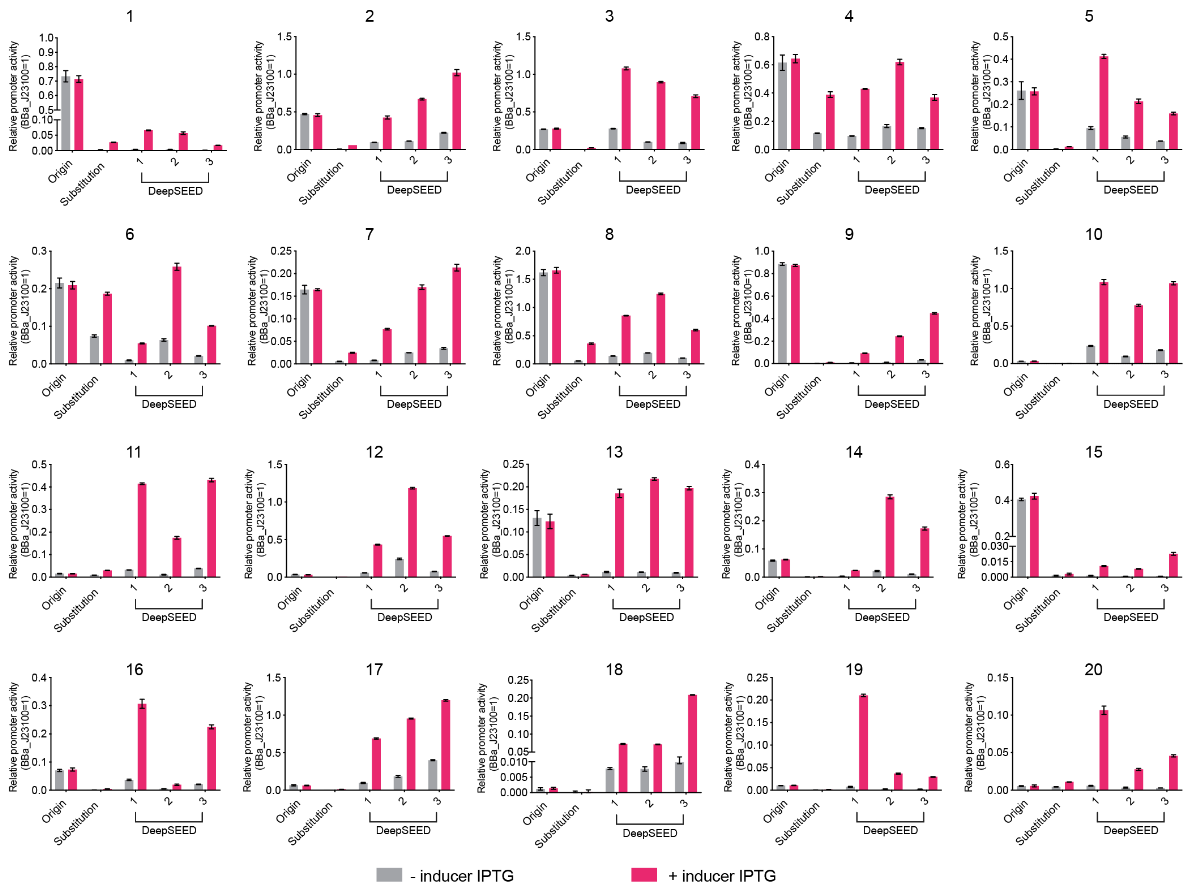

Supplementary Figure 14. The expression level of the 3lacO promoters in the IPTG-inducible promoter task in *E. coli*. The details of each promoter activity under the induced or non-induced conditions. The error bar represents the standard deviation of three biological replicates. Source data are provided as a Source Data file.

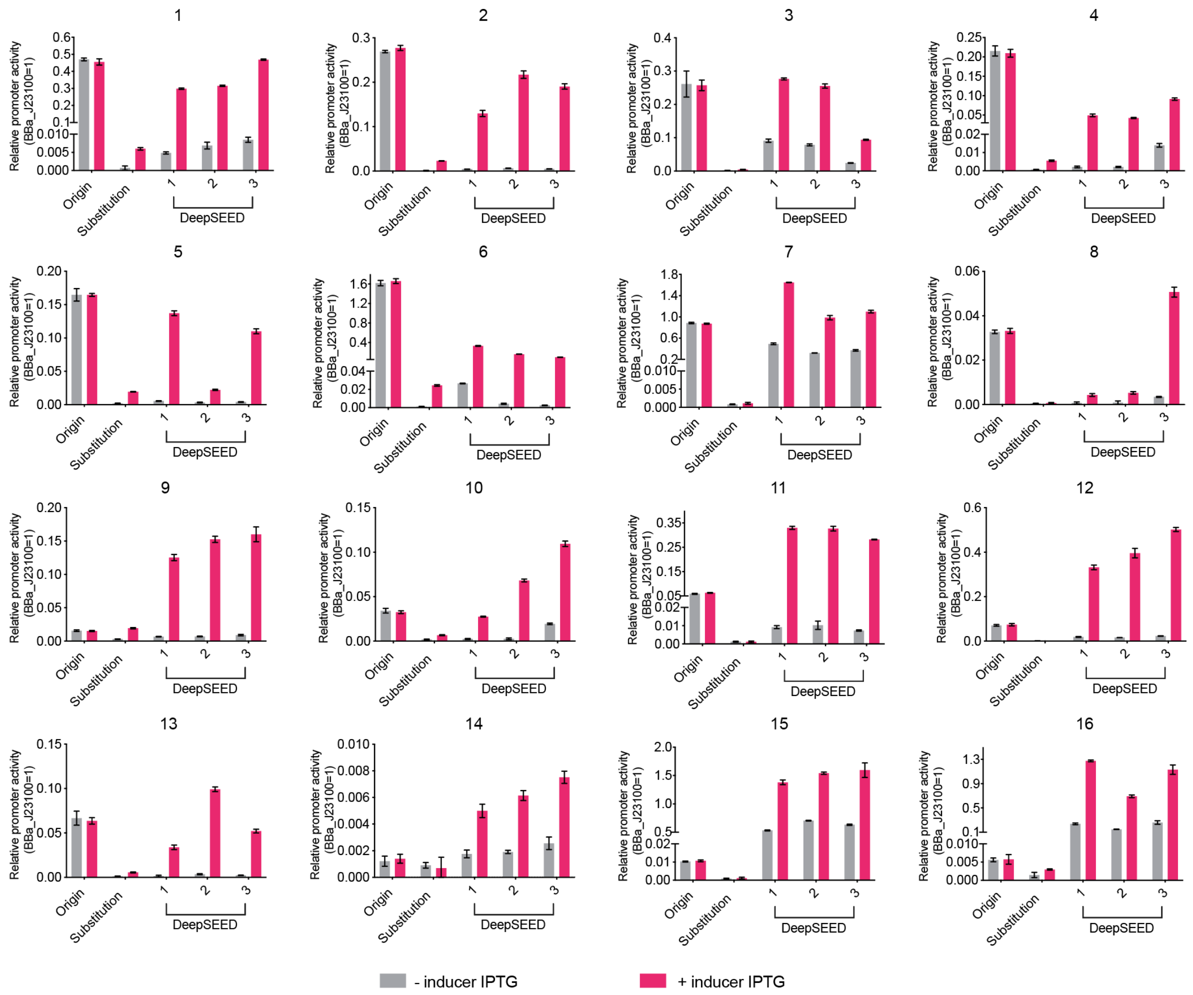

Supplementary Figure 15. The expression level of the 4lacO promoters in the IPTG-inducible promoter task in *E. coli*. The details of each promoter activity under the induced or non-induced conditions. The error bar represents the standard deviation of three biological replicates. Source data are provided as a Source Data file.

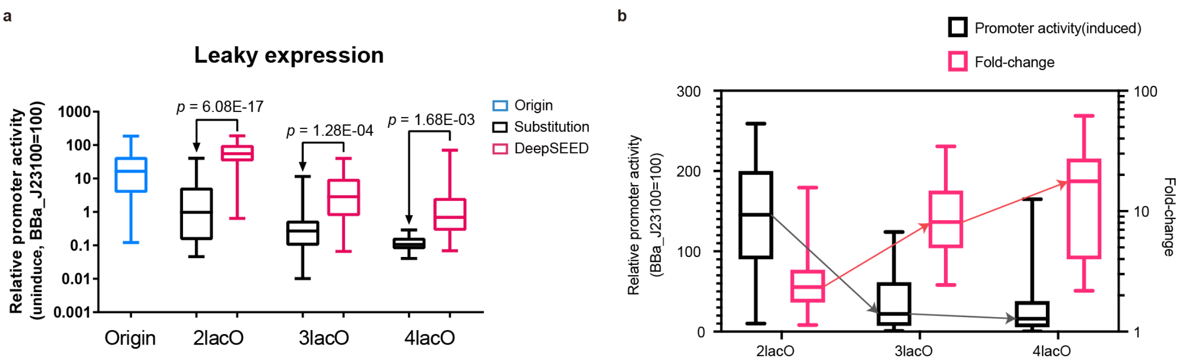

Supplementary Figure 16. a Boxplot showing the leaky expression of the Fig. 4b promoters *in vivo*. Normalization of promoter activity with strong constitutive promoter J23100 from the iGEM parts registry. Comparison of the leaky expression between the DeepSEED and substitution promoters. *P* values were determined by two-tailed unpaired Welch's *t*-test. **b** The performance of the DeepSEED-generated promoters in the IPTG-inducible promoter design task. Arrows show the direction in which promoter performance changes with the number of lacO sites. The result comes from Fig. 4c. Box plots indicate the median (middle line), 25th, 75th percentile (box) and minimum and maximum values (whiskers). Source data are provided as a Source Data file.

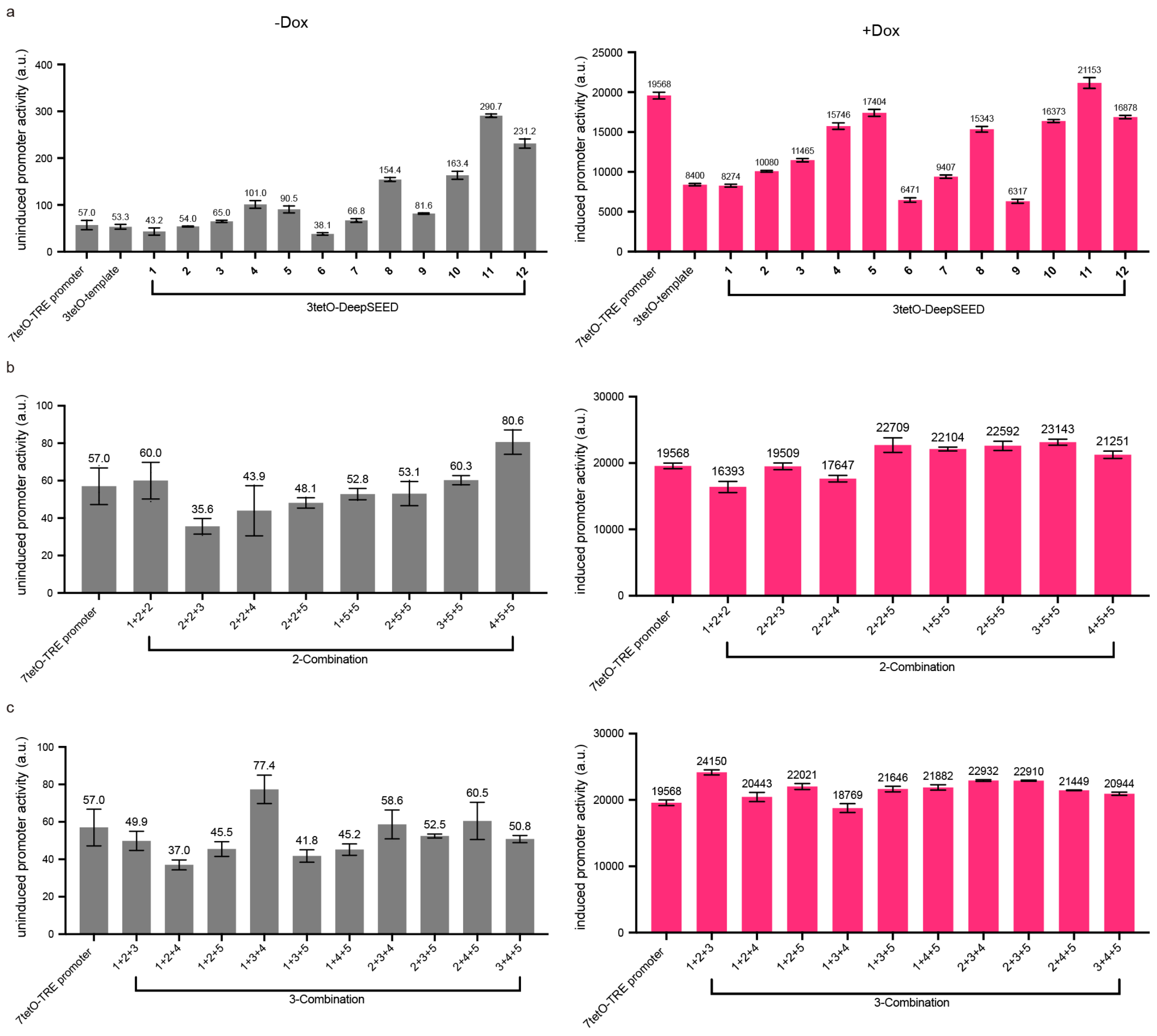

Supplementary Figure 17. The expression level of the promoters in the Dox-inducible promoter task in HEK293 cells. The details of each promoter activity under the induced and non-induced conditions. **a** 3tetO-DeepSEED promoters. **b** 2-Combination promoters **c** 3-Combination promoters. The error bar represents the standard deviation of three biological replicates. Source data are provided as a Source Data file.

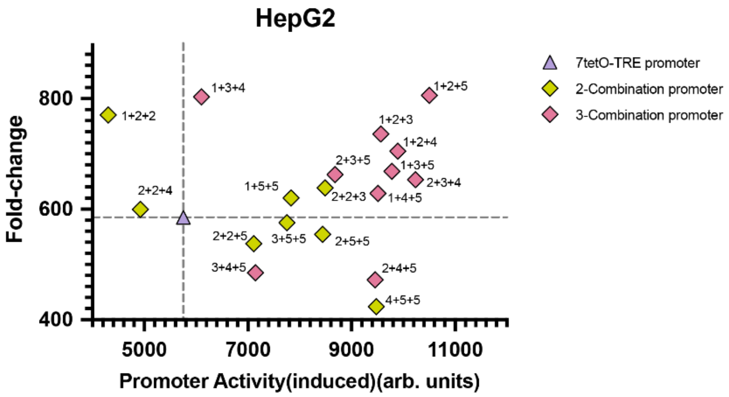

Supplementary Figure 18. Two-dimensional images showing the induced promoter activity and fold-change of the promoters in Fig. 5d, which were tested in the HepG2 cell line. Each dot represents the average of three biological replicates. Source data are provided as a Source Data file.

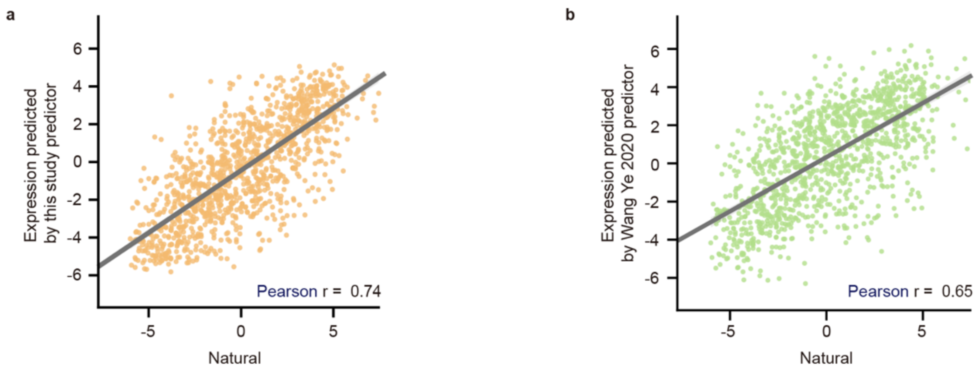

Supplementary Figure 19. A Scatter plot between real and DeepSEED predicted expression level in *E. coli* training dataset. **b** Scatter plot between real and CNN-based[4] predictive model predicted expression level in *E. coli* training dataset.

Supplementary References

1. He, K., Zhang, X., Ren, S. & Sun, J. Deep Residual Learning for Image Recognition. *arXiv [cs.CV]* (2015) doi:10.48550/ARXIV.1512.03385.
2. Huang, G., Liu, Z., van der Maaten, L. & Weinberger, K. Q. Densely Connected Convolutional Networks. *arXiv [cs.CV]* (2016).
3. Vaswani, A. *et al.* Attention Is All You Need. *arXiv [cs.CL]* (2017) doi:10.48550/ARXIV.1706.03762.
4. Wang, Y. *et al.* Synthetic promoter design in Escherichia coli based on a deep generative network. *Nucleic Acids Res.* **48**, 6403–6412 (2020).
5. Johns, N. I. *et al.* Metagenomic mining of regulatory elements enables programmable species-selective gene expression. *Nat. Methods* **15**, 323–329 (2018).
6. Wang, J. *et al.* HACER: an atlas of human active enhancers to interpret regulatory variants. *Nucleic Acids Res.* **47**, D106–D112 (2019).
7. Ernst, J. *et al.* Genome-scale high-resolution mapping of activating and repressive nucleotides in regulatory regions. *Nat. Biotechnol.* **34**, 1180–1190 (2016).
8. Castro-Mondragon, J. A. *et al.* JASPAR 2022: the 9th release of the open-access database of transcription factor binding profiles. *Nucleic Acids Res.* **50**, D165–D173 (2022).
9. Alper, H., Fischer, C., Nevoigt, E. & Stephanopoulos, G. Tuning genetic control through promoter engineering. *Proc. Natl. Acad. Sci. U. S. A.* **102**, 12678–12683 (2005).
10. Smolke, C. D. Building outside of the box: iGEM and the BioBricks foundation. *Nat. Biotechnol.* **27**, 1099–1102 (2009).
11. Yu, T. C. *et al.* Multiplexed characterization of rationally designed promoter architectures deconstructs combinatorial logic for IPTG-inducible systems. *Nat. Commun.* **12**, 325 (2021).
