# Supplementary material for: Deep flanking sequence engineering for efficient promoter design using DeepSEED
Source: Nat Commun. 2023 Oct 9;14:6309. doi: 10.1038/s41467-023-41899-y (PMC10562447; doi:10.1038/s41467-023-41899-y)
Supplement: Supplementary file 3 — Description of Additional Supplementary Files Document [file 41467_2023_41899_MOESM3_ESM.pdf]

Description of Additional Supplementary files

File Name: Supplementary Data 1

Description: All the promoter sequences oligos synthesized in this study and their activity measured by plate reader or flow cytometry.

File Name: Supplementary Data 2

Description: All the plasmid sequences used in this study.

File Name: Supplementary Data 3

Description: Promoter sequences and results for edit distance analysis and BLAST.
