# Supplementary material for: Deep flanking sequence engineering for efficient promoter design using DeepSEED
Source: Nat Commun. 2023 Oct 9;14:6309. doi: 10.1038/s41467-023-41899-y (PMC10562447; doi:10.1038/s41467-023-41899-y)
Supplement: Supplementary file 5 — Reporting Summary [file 41467_2023_41899_MOESM5_ESM.pdf]

nature portfolio

Corresponding author(s): Xiaowo Wang

Last updated by author(s): Aug 31, 2023

Reporting Summary

Nature Portfolio wishes to improve the reproducibility of the work that we publish. This form provides structure for consistency and transparency in reporting. For further information on Nature Portfolio policies, see our Editorial Policies and the Editorial Policy Checklist.

Statistics

For all statistical analyses, confirm that the following items are present in the figure legend, table legend, main text, or Methods section.

n/a	Confirmed	
☐	☒	The exact sample size (*n*) for each experimental group/condition, given as a discrete number and unit of measurement
☐	☒	A statement on whether measurements were taken from distinct samples or whether the same sample was measured repeatedly
☐	☒	The statistical test(s) used AND whether they are one- or two-sided<br>*Only common tests should be described solely by name; describe more complex techniques in the Methods section.*
☐	☒	A description of all covariates tested
☐	☒	A description of any assumptions or corrections, such as tests of normality and adjustment for multiple comparisons
☐	☒	A full description of the statistical parameters including central tendency (e.g. means) or other basic estimates (e.g. regression coefficient) AND variation (e.g. standard deviation) or associated estimates of uncertainty (e.g. confidence intervals)
☐	☒	For null hypothesis testing, the test statistic (e.g. *F*, *t*, *r*) with confidence intervals, effect sizes, degrees of freedom and *P* value noted<br>*Give P values as exact values whenever suitable.*
☒	☐	For Bayesian analysis, information on the choice of priors and Markov chain Monte Carlo settings
☒	☐	For hierarchical and complex designs, identification of the appropriate level for tests and full reporting of outcomes
☐	☒	Estimates of effect sizes (e.g. Cohen's *d*, Pearson's *r*), indicating how they were calculated

Our web collection on statistics for biologists contains articles on many of the points above.

Software and code

Policy information about availability of computer code

Data collection

96-well microplates were analyzed using SkanIt Software 2.4.5 RE for Varioskan Flash
Flow cytometry data was gathered using BD FASCDiva Software(8.0.1)

Data analysis

DNA shapes (MGW, Roll, ProT and HelT) of promoter sequence was calculated by DNAshape (Version 2.6.0).
Location of potential second promoter in E.coli promoter sequences were found by biopython package (Version 1.7.9).
Identification of TFBSs at eukaryotic promoters was performed by FIMO (MEME Version 5.1.0) when inputting their corresponding position frequency matrices (PFM).
For optimizing promoter sequences by genetic algorithm, Scikit-opt (Version 0.6.5) was used.
The second stage of calculating the semantic sequence space was performed by T-SNE dimension reduction (MulticoreTSNE Version 0.1.0)
DeepSEED was implemented and trained in pytorch (Version 1.7.1+cu110). Custom code used in this manuscript are available on Github. (https://github.com/WangLabTHU/deepseed).
96-well microplates data was analyzed using Excel (Version 16.59) and Prism(Version 9.0.0). Flow cytometry data was analyzed using FlowJo (Version 10.8.1), Excel (Version 16.59) and Prism(Version 9.0.0).
Blastn search was operated on the experimental validated sequences via website (https://blast.ncbi.nlm.nih.gov/Blast.cgi?PROGRAM=blastn&BLAST_SPEC=GeoBlast&PAGE_TYPE=BlastSearch)

For manuscripts utilizing custom algorithms or software that are central to the research but not yet described in published literature, software must be made available to editors and reviewers. We strongly encourage code deposition in a community repository (e.g. GitHub). See the Nature Portfolio guidelines for submitting code & software for further information.

Data

Policy information about availability of data

All manuscripts must include a data availability statement. This statement should provide the following information, where applicable:

- Accession codes, unique identifiers, or web links for publicly available datasets
- A description of any restrictions on data availability
- For clinical datasets or third party data, please ensure that the statement adheres to our policy

The promoter sequences generated and tested in this study are available in the Supplementary Data 1 and 3. The plasmid sequences used in this study are provided in the Supplementary Data 2. Source data are provided with this paper. This work utilized several published datasets. Datasets proposed by Ernst et al. were used to construct the training set of the predictor in eukaryotic promoter design (https://www.ncbi.nlm.nih.gov/geo/query/acc.cgi?acc=GSE71279). Enhancer datasets of HEK293 cell line called HACER were used to construct the training set of the generator in eukaryotic promoter design (http://bioinfo.vanderbilt.edu/AE/HACER/). Potential promoters in bacteria proposed by Johns et al. were used to construct the predictor and generator in prokaryotic promoter design (https://static-content.springer.com/esm/art%3A10.1038%2Fnmeth.4633/MediaObjects/41592_2018_BFnmeth4633_MOESM4_ESM.xlsx). The motif sequences in JASPAR database were used to find the potential binding sites in flanking sequence (https://jaspar.genereg.net/).

Research involving human participants, their data, or biological material

Policy information about studies with human participants or human data. See also policy information about sex, gender (identity/presentation), and sexual orientation and race, ethnicity and racism.

Reporting on sex and gender	N/A
Reporting on race, ethnicity, or other socially relevant groupings	N/A
Population characteristics	N/A
Recruitment	N/A
Ethics oversight	N/A

Note that full information on the approval of the study protocol must also be provided in the manuscript.

Field-specific reporting

Please select the one below that is the best fit for your research. If you are not sure, read the appropriate sections before making your selection.

☒ Life sciences ☐ Behavioural & social sciences ☐ Ecological, evolutionary & environmental sciences

For a reference copy of the document with all sections, see nature.com/documents/nr-reporting-summary-flat.pdf

Life sciences study design

All studies must disclose on these points even when the disclosure is negative.

Sample size	No special sample size calculations were performed. The models were trained on a diverse range of publicly accessible datasets. Because the evaluation of deep learning models was conducted directly using separate test sets, without making any assumptions about underlying distributions, there was no need rely on theoretical sample size. By convention, three biological replicates are used to determine the activity of promoter sequences.
Data exclusions	No data were excluded from the analysis
Replication	The activity of the promoter sequences was measured in three biological replicates. The correlations among replicates are high and all of them were successful.
Randomization	This is not relevant to our study. Promoters activity were measured in vivo. We chose three short promoters with different activities as template sequences for constitutive promoter design. We randomly chose the original sequence from the MPRA dataset for IPTG-inducible promoter design. We chose the wild-used TRE promoter as the template sequence for the Dox-inducible promoter design.
Blinding	All experiments were done in cell culture, therefore blinding did not apply. Groups were not allocated in this work and conclusions were determined through objective and statistical analyses.

Reporting for specific materials, systems and methods

We require information from authors about some types of materials, experimental systems and methods used in many studies. Here, indicate whether each material, system or method listed is relevant to your study. If you are not sure if a list item applies to your research, read the appropriate section before selecting a response.

Materials & experimental systems

n/a	Involved in the study
[x]	[ ] Antibodies
[ ]	[x] Eukaryotic cell lines
[x]	[ ] Palaeontology and archaeology
[x]	[ ] Animals and other organisms
[x]	[ ] Clinical data
[x]	[ ] Dual use research of concern
[x]	[ ] Plants

Methods

n/a	Involved in the study
[x]	[ ] ChIP-seq
[ ]	[x] Flow cytometry
[x]	[ ] MRI-based neuroimaging

Eukaryotic cell lines

Policy information about cell lines and Sex and Gender in Research

Cell line source(s)	HEK293 (293-H, from Invitrogen) and HepG2 (from the Institute of Basic Medical Sciences of CAMS) cell lines used in our study.
Authentication	HEK293(293-H) was authenticated upon purchased from Invitrogen (STR profile validated); HepG2 cell line was authenticated upon purchased from the Institute of Basic Medical Sciences of CAMS (STR profile validated).
Mycoplasma contamination	Cell lines were tested negative for mycoplasma contamination at the time of purchase. The cell line did not exhibit any abnormal features during subsequent culture.
Commonly misidentified lines (See ICLAC register)	No commonly misidentified cell lines were used.

Flow Cytometry

Plots

Confirm that:

[x] The axis labels state the marker and fluorochrome used (e.g. CD4-FITC).

[x] The axis scales are clearly visible. Include numbers along axes only for bottom left plot of group (a 'group' is an analysis of identical markers).

[x] All plots are contour plots with outliers or pseudocolor plots.

[x] A numerical value for number of cells or percentage (with statistics) is provided.

Methodology

Sample preparation	Cells were trypsinized 24 h after transfection and were then centrifuged at 300 × g for 5 min at room temperature. Then, the cells were washed with phosphate-buffered saline (PBS) once and resuspended in 1x PBS in a total volume of 400 µl. Next, the cells were analyzed using LSRFortessa (BD Biosciences). The excitation lasers (Ex), emission filters (Em), and photomultiplier tube (PMT) voltage used for respective fluorescent protein measurements are as follows: TagBFP (Ex: 405 nm laser, Em: 450/50 filter, PMT: 350 V for HEK293; 300V for HepG2). EYFP (Ex: 488 nm laser, Em: 530/30 filter, PMT: 200 V). For each sample, ?1*105 cell events were collected for downstream analysis.
Instrument	LSRFortessa (BD Biosciences)
Software	Collect: BD FASCDiva Software<br>Analyze: FlowJo(10.8.1)
Cell population abundance	No cell sorting was performed in this work
Gating strategy	All populations were gated by FSC-A/SSC-A to isolate the . Singlets were gated in FSC-W/FSC-H and SSC-W/SSC-H. Cells with a TagBFP intensity between 1*104~5*104 (HEK293 cells) and 8*102~5*103 (HepG2 cells) were selected containing the proper concentration of rtTA proteins. The mean of EYFP as the activity of the designed promoters.

[ ] Tick this box to confirm that a figure exemplifying the gating strategy is provided in the Supplementary Information.
